# Insecticide resistance status in *Anopheles gambiae *in southern Benin

**DOI:** 10.1186/1475-2875-9-83

**Published:** 2010-03-24

**Authors:** Anges W Yadouleton, Gil Padonou, Alex Asidi, Nicolas Moiroux, Sahabi Bio-Banganna, Vincent Corbel, Raphael N'guessan, Dina Gbenou, Imorou Yacoubou, Kinde Gazard, Martin C Akogbeto

**Affiliations:** 1 Centre de Recherche Entomologique de Cotonou (CREC), 06 BP 2604 Cotonou, République du Bénin; 2 Institut de Recherche pour le Développement (IRD), UR016, Caractérisation et Contrôle des Populations de Vecteurs, 01 BP 4414 RP Cotonou, République du Bénin; 3 London School of Tropical Medicine and Hygiene, London, UK; 4 WHO-Benin; 5 National Malaria Control Programme, Benin; 6 Faculté des Sciences de la Santé, Benin

## Abstract

**Background:**

The emergence of pyrethroid resistance in *Anopheles gambiae *has become a serious concern to the future success of malaria control. In Benin, the National Malaria Control Programme has recently planned to scaling up long-lasting insecticidal nets (LLINs) and indoor residual spraying (IRS) for malaria prevention. It is, therefore, crucial to monitor the level and type of insecticide resistance in *An. gambiae*, particularly in southern Benin where reduced efficacy of insecticide-treated nets (ITNs) and IRS has previously been reported.

**Methods:**

The protocol was based on mosquito collection during both dry and rainy seasons across forty districts selected in southern Benin. Bioassay were performed on adults collected from the field to assess the susceptibility of malaria vectors to insecticide-impregnated papers (permethrin 0.75%, delthamethrin 0.05%, DDT 4%, and bendiocarb 0.1%) following WHOPES guidelines. The species within *An. gambiae *complex, molecular form and presence of *kdr and ace-*1 mutations were determined by PCR.

**Results:**

Strong resistance to permethrin and DDT was found in *An. gambiae *populations from southern Benin, except in Aglangandan where mosquitoes were fully susceptible (mortality 100%) to all insecticides tested. PCR showed the presence of two sub-species of *An. gambiae*, namely *An. gambiae s.s*, and *Anopheles melas*, with a predominance for *An. gambiae s.s *(98%). The molecular M form of *An. gambiae *was predominant in southern Benin (97%). The *kdr *mutation was detected in all districts at various frequency (1% to 95%) whereas the *Ace-1 *mutation was found at a very low frequency (≤ 5%).

**Conclusion:**

This study showed a widespread resistance to permethrin in *An. gambiae *populations from southern Benin, with a significant increase of *kdr *frequency compared to what was observed previously in Benin. The low frequency of *Ace-1 *recorded in all populations is encouraging for the use of bendiocarb as an alternative insecticide to pyrethroids for IRS in Benin.

## Background

More than 90% of recorded malarial deaths occur in Africa among the most vulnerable low immune response individuals, such as children under five years old and pregnant women [1,2]. The National Malaria Control Programmes (NMCP) in African countries currently relies on strategies targeting mosquito vector control, which involve the use of long-lasting insecticidal nets (LLINs) and/or indoor residual spraying (IRS), the two most effective preventive measures. Both methods have shown to be very effective against *Anopheles *mosquitoes [3-8]. In 2010 in Benin, a full coverage of LLINs countrywide couple with IRS in the department of Ouémé in southern Benin will become a new tool to improve malaria prevention and control.

However, the development of pyrethroid resistance in populations of *Anopheles gambiae *has become a serious threat to the effectiveness of these two vector control measures [9]. N'Guessan *et al *[10] recently established a clear relationship between pyrethroid resistance caused by *kdr *and the failure of LLINs and IRS in experimental huts in south Benin. In the last decade, the emergence of resistance in populations of *Anopheles *to common classes of insecticides used in public health has been reported in many African countries including Kenya [11], Côte d'Ivoire [12], Benin [13-15], Niger [16], Burkina Faso [17,18], Mali[19], Nigeria[20], South Africa [21], and Cameroun [22].

In West Africa, the main mechanism involved in pyrethroid-resistance in *An. gambiae *is caused by target site insensitivity through a knockdown resistance (*kdr*)-like mutation caused by a single point mutation (*Leu-Phe*) in the para-sodium channel gene [23]. Preliminary surveys done in Benin southern in *An. gambiae *populations by Corbel et *al *[14], indicated that the *Leu-Phe kdr *mutation has been found almost only in the M form at high frequency (0.95).

Indeed, several authors reported that the use of insecticides in households and pesticides in agricultural settings has greatly increased selection pressure leading to the emergence of insecticide resistance in malaria vectors [13-15,24]. In Benin, it was reported that DDT resistance in *An. gambiae sensu lato (s.l.) *was the result of massive use of DDT house spraying applications in several districts of the country during the WHO malaria eradication campaign in the 1950s [25].

A study in Burkina Faso documented a relatively high frequency of *kdr *mutations (*Leu-Phe) *in *An. gambiae *collected from cotton farms under massive insecticide treatment compared to farms with no pesticide utilization [18]. Therefore, several studies on insecticide resistance have currently been addressing the agenda of most malaria scientists in sub-Saharan Africa and around the world to approach in different ways the crucial issues of insecticide resistance in malaria vectors which threatens the effective usefulness of ITNs and IRS for malaria prevention [6,26].

Previous resistance monitoring surveys conducted in Benin had focused on the south-north transect. It is then a priority to investigate the status of insecticide resistance in *An. gambiae *in southern Benin, because pyrethroid resistance has been reported with a clear evidence of reduced efficacy of ITNs and IRS in experimental huts [10,26]. In addition to the *kdr *mutation, which is the main mechanism of resistance to pyrethroids, it's important to address also the distribution of the *Ace.1 *allele that causes resistance to organophosphates and carbamates. With support of Presidential Malaria Initiative (PMI), a large-scale programme based on free-ITN distribution in combination with IRS was implemented since 2008 in four districts in Department of Ouémé in southern Benin. To attain a better understanding of the resistance situation in Benin particularly in these localities because of the use of bendiocarb in IRS, it is important to characterize the spatial distribution of resistance in *An. gambiae *in a variety of ecological settings and then attempt to correlate this resistance with pesticide usage. The present study propose to assess the resistance status of malaria vectors to carbamates, pyrethroids and assessed the implications for vector control strategy in a new geographical setting of an opposite east-west transect of the southern part of Benin. This area has a different bioclimatic characteristic with high rainfall (1,500 mm yearly), where insecticides are extensively used for both public health and agricultural purposes.

## Methods

### Study areas

The study was carried out in forty districts of southern Benin characterized by a continual practice of urban and peri-urban agriculture, with two rainy seasons (March- July and October- November) and two dry seasons (December-March and August-September). The annual mean rainfall is 1,500 mm in July, relative humidity (RH) of 70% ± 5 and a minimum/maximum temperature ranging from 23 to 32°C. The choice of these environments is justified by their particular bioclimatic characteristics and the use of insecticides or fertilizers in public health and agriculture. Indeed, the presence of susceptible population of *An. gambiae *to pyrethroids and organophosphorous recorded in some districts will help to effectively use the IRS and ITNs in the study areas.

### Mosquito collections

Mosquitoes were collected during the dry (from February to March) and the rainy seasons (April-July) across the forty districts selected in south Benin. Larvae and pupae were collected using the dipping on breeding sites and then kept in separated labelled bottles related to each locality. A part of the larvae samples was reared up to adult emergence at the CREC (Centre de Recherche Entomologique de Cotonou, Benin) insectary for further bioassay tests.

### Insecticide susceptibility test

Females mosquitoes aged 2-5 days old were exposed to diagnostic doses of various insecticides for susceptibility tests using insecticide-impregnated papers, as described by the standard WHO testing protocol [27]. The following insecticides were tested: deltamethrin (0.05%), permethrin (0.75%), DDT (4%) and bendiocarb (0.1%). The emphasis was also put on deltamethrin, because of a nationwide distribution of PermaNets by the NMCP. The use of DDT is justified by the detection of cross-resistance between pyrethroids and organo-chlorine in *Anopheles *populations. The carbamate bendiocarb was one of the alternative insecticides to pyrethroids currently used for IRS in Benin [28].

For each treatment, five test tubes were used: one untreated paper as a control and four treated papers to expose mosquitoes. Control tubes contained filter papers impregnated with silicon oil (insecticide carrier) only, whereas treated papers were impregnated with diagnostic doses of insecticide plus carrier.

An average of twenty-five mosquitoes was introduced into each tube. Females of *An. gambiae *used in this study were exposed for one hour to insecticide-treated papers and monitored at different time intervals (10, 15, 20, 30, 45, 60 minutes) to record the "knock-down" times. After one-hour exposure, mosquitoes were transferred into holding tubes and provided with cotton wool wetted with a 10% honey solution. Mortalities were recorded after 24 hours and the susceptibility status of the population was graded according to the WHO recommended protocol [27]. Dead and survived mosquitoes from this bioassay were separately kept in Carnoy solution at -20°C for further molecular characterization.

### Molecular characterization of Anopheles populations using PCR analysis

In each locality, 25-35 females of *An. gambiae *samples from the WHO bioassays were analysed at the molecular level. PCR analysis for species identification [29] was performed to identify various members of *An. gambiae *complex collected in each site. The next set of PCR focused on molecular forms using PCR-RFLP [30], which involved only *An. gambiae sensu stricto (s.s.)*. The PCR forms sub-grouped the *An. gambiae s.s*. into two molecular forms: *An. gambiae s.s. M *and *An. gambiae s.s. S *forms. The last series of PCRs determined the presence of *kdr *mutations in *An. gambiae ss*. Populations, as described by Martinez-Torres *et al *[31]. The PCR-RFLP diagnostic test was used to detect the presence of G119S mutation (*Ace.1 *gene) as described by Weill *et al *[32].

### Data interpretation

The resistant status of mosquito samples was determined according to the WHO criteria [27].

Following the WHO protocol

- Mortality rates is > 97%: the population was considered fully susceptible

- Mortality rates ranged between 80 > × < 97%: resistance suspected in the population

- Mortality rates < 80%, the population was considered resistant to the tested insecticides.

Mortality rates were corrected using Abbott formula when control mortality was above 5% [33]. Molecular results (PCR *Kdr and Ace-1*) were compared to insecticide susceptibility tests performed with the WHO method to conclude on the *An. gambiae *status in the districts surveyed.

### Mapping insecticide resistance in mosquito vectors in southern Benin

Using geographical information recorded with GPS, screened localities were projected on a map of southern Benin where mosquito larvae were collected. Resistance data were obtained from 40 districts in 6 departments (Ouémé, Plateau, Littoral, Atlantique, Mono, Couffo) and spatialized using the ESRI ArcGis Software.

### Data analysis

Analysis using the computer software Excel, Fisher's exact tests was performed on the data sets gathered from the localities surveyed. Parameter for analysis included the resistance status of each tested population of *An. gambiae*. The insecticide susceptibility test on resistant strains from different districts was compared and analysed using Stat-calc-Epi-info software, to compare the status of insecticide resistance in the different sites investigated. A Fisher's exact test was performed to determine if there was any significant difference between two given sites.

## Results

### Resistance status

Additional file 1 shows the insecticide resistance status of *An. gambiae s.l *populations from the 40 districts of southern Benin. Following the exposure of females of *An. gambiae *to permethrin impregnated papers, all 40 populations were fully susceptible to deltamethrin and bendiocarb, 39 out of 40 were resistant to permethrin and 39 out of 40 showed resistant to DDT (see Additional file 1)

### Identification of molecular forms of *Anopheles gambiae *s.s

1,500 mosquitoes from the 40 districts were successfully analysed by species, molecular forms. PCR revealed the presence of two sub-species of *An. gambiae: An. gambiae s.s., and Anopheles melas *with a predominance of *An. gambiae s.s *(98%). The M form was predominant over the S form (M = 98%; S = 2%).

### Detection of resistance genes

Allele and genotype frequencies at the *kdr *and *Ace.1 *loci are shown in Additional file 2. Results from this study showed that the *kdr *mutation was present in all *An. gambiae *populations collected from the different district. The highest frequencies were recorded in Dogbo, Lokossa and Lanta (96%, 95% and 95%, respectively) and the lowest frequency was recorded in *An. gambiae *strains from Aglandan (1%). The *Ace-1 *mutation was found in *An. gambiae *populations collected from the different districts but at very low frequency (from 1% to 5%).

### Mapping insecticide resistance in mosquito vectors in southern Benin

**Figure 1:**
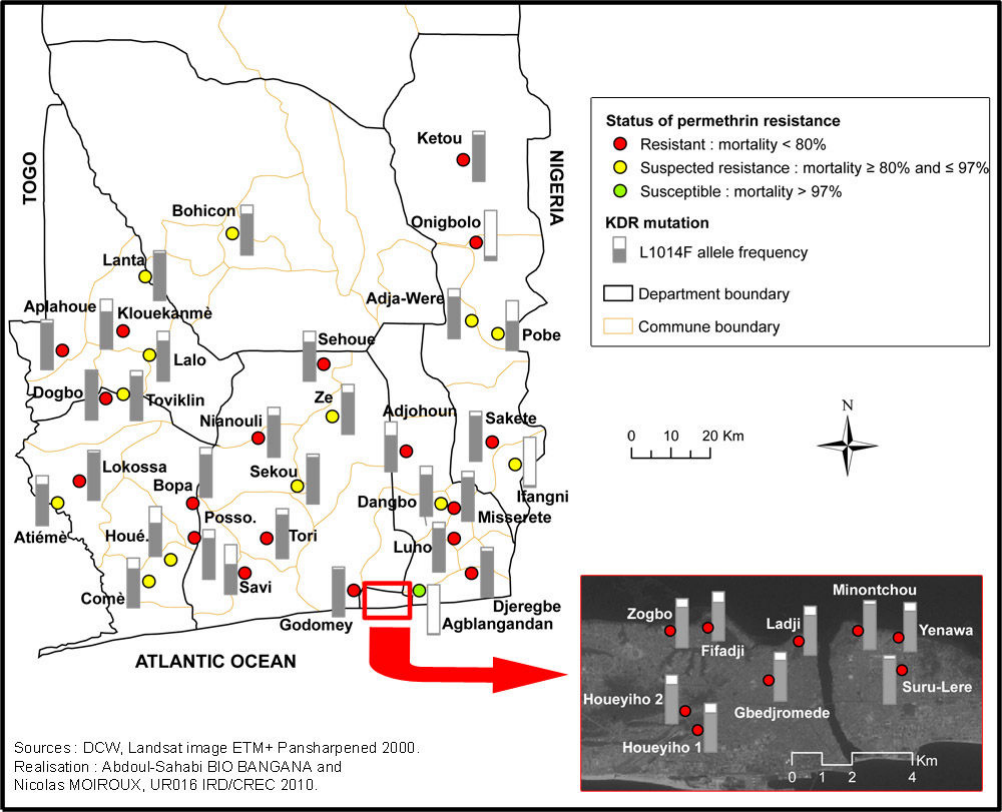
**Distribution of *Anopheles gambiae *resistance/susceptibility to permethrin (0.75%) in southern Benin**.

Using geographical information recorded with GPS, screened localities were projected on a map of Benin and areas of permethrin and bendiocarb resistance or susceptibility were marked. The map generated from this study showed a spread of resistance of permethrin in most districts of South Benin (Figure 1). However, the levels of resistance registered in the following localities, Sakété, Kétou, Lokossa, Dogbo, Comè and Lanta were relatively consistent compared to other sites of the districts. On the other hand, no resistance was found in, Houeyogbé, Aglangandan, and Ifangni, districts.

## Discussion

The species composition of *Anopheles gambiae *complex identified during this study did not differ from those recorded in previous studies in Benin. It was shown that, within the *An. gambiae *complex, *An. gambiae *s.s. M form was predominant (> 98%) and has as a wide distribution across southern Benin Corbel *et al *[14]. The same trend was found in some localities of Mali, Nigeria [34-36]. The absence of *An. melas *in many districts in southern Benin can be attributed to the fact that larvae were mostly sampled from pools and puddles rather than flooded water, which is the preferred breeding site of *An. melas *[37]. No *An. arabiensis *was found in the different localities.

The study showed a wide distribution of resistance in *An. gambiae s.l*. to permethrin and DDT in southern Benin whereas all samples of *An. gambiae *tested were fully susceptible to deltamethrin and bendiocarb. The widespread resistance to DDT and permethrin in southern Benin can be explained by a long-standing, massive use of DDT house-spraying in several districts of the country during the WHO malaria eradication programme in the 1950s [25]. In addition, the rapid expansion of urban agriculture is one of the major factors that contributes to a large distribution of pyrethroid resistance in *An. gambiae s.l *Corbel *et al *[14]. A recent report by Yadouleton *et al *[15] has confirmed that urban farming in Benin has enormously contributed to the emergence of resistance in *Anopheles *populations. As reported by Akogbeto *et al *[24], some populations of *An. gambiae *lay their eggs in breeding sites containing insecticide residues. A study in vegetable farming in Benin [15] has shown that such activities in urban areas directly led to an improper use of insecticides to control vegetable pests, thus exerting a huge selection pressure on mosquito larval population. Moreover, the liberalization of the pesticide sector and the increased cost of pesticides registration have incited most of the farmers to illegally procure insecticides and an uncontrolled use of these chemicals in Benin. This factor has also contributed to the emergence of insecticide resistance in *An. gambiae *populations [15,24].

However, the absence of pyrethroid-DDT cross resistance in *An. gambiae *from Aglangandan in the Departement of Ouémé can be explained by the absence of agriculture activities in this area. Recently, qualitative data were collected from direct observations, in-depth interview and focus group discussions to confirm this hypothesis [15].

Some populations of *An. gambiae *have developed low resistance to bendiocarb in southern Benin. This can be explained by the fact that in these areas, carbamate and organophosphorous insecticides were mostly used by farmers for crop protection [38]. The results confirm those of Corbel et *al *[14] and Djogbenou et *al *[39] that previously showed a low frequency of the Ace1 allele in malaria vectors populations in Benin. This is particularly relevant to strengthen vector control campaigns using Indoor Residual Spraying based on carbamate and/or organophosphate as alternatives to pyrethroids, which are currently used by the NMCP in several areas of Benin.

## Conclusion

The emergence of pyrethroid resistance in *An. gambiae *has become a serious concern for the success of malaria control in the last decades. To date, pyrethrinoids remain the only family of insecticides currently recommended by the WHO for the impregnation of bed nets. This study showed a relatively wide distribution of insecticide resistance in *An. gambiae*, especially to permethrin and DDT. The current findings will help for decision-making in the National Malaria Control Programme especially in the choice of insecticide to use during the next campaigns of Indoor Residual Spraying (IRS) in Benin.

## Competing interests

The authors declare that they have no competing interests.

## Authors' contributions

AWY contributed to design of the study and conceived the protocol, proceed data analysis and interpretation. MCA, DG, KD, IY contributed to the study design, provided funding and coordination. GP, contributed in the study design and in the implementation of this research. BB and NM contributed to the mapping. AA, NR, VC contributed to manuscript drafting. All authors read and approved the final manuscript.

## Supplementary Material

Additional file 1 Percentage of dead *Anopheles gambiae *observed after 1 hour exposure to permethrin (0.75%), bendiocarb (0.1%), DDT (4%), deltamethrin (0.05%) in 6 departments in southern Benin. Click here for file

Additional file 2 Species identification, molecular forms and frequency of the *kdr*, and *Ace.1 *alleles and genotypes in *Anopheles gambiae s.l* Click here for file
