# Supplementary material for: Insecticide resistance status in Anopheles gambiae in southern Benin
Source: Malar J. 2010 Mar 24;9:83. doi: 10.1186/1475-2875-9-83 (PMC2858214; doi:10.1186/1475-2875-9-83)
Supplement: Additional file 1 — Percentage of dead Anopheles gambiae observed after 1 hour exposure to permethrin (0.75%), bendiocarb (0.1%), DDT (4%), deltamethrin (0.05%) in 6 departments in southern Benin. [file 1475-2875-9-83-S1.DOC]

**Additional file 1**: **Percentage of dead *Anopheles gambiae* observed after 1 hour exposure to permethrin (0.75%), bendiocarb (0.1%), DDT (4%), deltamethrin (0.05%) in 6 departments in southern Benin.**

|  |  | **Permethrin** | | | **Delthamethrin** | | | **DDT** | | | **Bendiocarb** | | |
| --- | --- | --- | --- | --- | --- | --- | --- | --- | --- | --- | --- | --- | --- |
| **Departments** | **Localities** | **N tested** | **% Mortality** | **Status** | **N tested** | **% Mortality** | **Status** | **N tested** | **% Mortality** | **Status** | **N tested** | **% Mortality** | **Status** |
|  | Dogbo | 100 | 54^a^ | R | 100 | 100 | S | 100 | 45.1 ^a^ | R | 100 | 100 | S |
|  | Lokossa | 100 | 68.8 ^a^ | R | 100 | 100 | S | 100 | 30.7 ^a^ | R | 100 | 100 | S |
|  | Aplahoue | 100 | 78 ^a^ | R | 100 | 100 | S | 100 | 50 ^a^ | R | 100 | 100 | S |
|  | Houéyogbé | 100 | 85 ^a^ | R | 100 | 100 | S | 100 | 63.8 ^a^ | R | 100 | 100 | S |
|  | Lanta | 100 | 82.8 ^a^ | r | 100 | 100 | S | 100 | 30.9 ^a^ | R | 100 | 100 | S |
| Mono-Couffo | Klouekanmè | 100 | 75 ^a^ | R | 100 | 100 | S | 100 | 54 ^a^ | R | 100 | 100 | S |
|  | Atiémè | 100 | 80 ^a^ | R | 100 | 100 | S | 100 | 58 ^a^ | R | 100 | 100 | S |
|  | Toviklin | 100 | 83 ^a^ | r | 100 | 100 | S | 100 | 54 ^a^ | R | 100 | 100 | S |
|  | Lalo | 100 | 84 ^a^ | r | 100 | 100 | S | 100 | 54 ^a^ | R | 100 | 100 | S |
|  | Possotomè | 100 | 72 ^a^ | R | 100 | 100 | S | 100 | 45 ^a^ | R | 100 | 100 | S |
|  | Bopa | 100 | 75 ^a^ | R | 100 | 100 | S | 100 | 62 ^a^ | R | 100 | 100 | S |
|  | Comè | 100 | 80 ^a^ | R | 100 | 100 | S | 100 | 55 ^a^ | R | 100 | 100 | S |
|  | Pobe | 100 | 85 ^a^ | r | 100 | 100 | S | 100 | 48 ^a^ | R | 100 | 100 | S |
|  | Sakete | 100 | 56 ^a^ | R | 100 | 100 | S | 100 | 36 ^a^ | R | 100 | 100 | S |
| Ouémé-Plateau | Ifangni | 100 | 94.1 ^a^ | r | 100 | 100 | S | 100 | 61.9 ^a^ | R | 100 | 100 | S |
|  | Onigbolo | 100 | 78 ^a^ | R | 100 | 100 | S | 100 | 56 ^a^ | R | 100 | 100 | S |
|  | Adja-Were | 100 | 90 ^b^ | r | 100 | 100 | S | 100 | 56.7 ^a^ | R | 100 | 100 | S |
|  | Ketou | 100 | 75 ^a^ | R | 100 | 100 | S | 100 | 45 ^a^ | R | 100 | 100 | S |
|  | Dangbo | 100 | 83.8 ^a^ | r | 100 | 100 | S | 100 | 44.6 ^a^ | R | 100 | 100 | S |
|  | Adjohoun | 100 | 77.1 ^a^ | R | 100 | 100 | S | 100 | 54.2 ^a^ | R | 100 | 100 | S |
|  | Agblangandan | 100 | 100 ^b^ | S | 100 | 100 | S | 100 | 100 ^b^ | S | 100 | 100 | S |
|  | Misserete | 100 | 70.8 ^a^ | R | 100 | 100 | S | 100 | 38.7 ^a^ | R | 100 | 100 | S |
|  | Djeregbe | 100 | 64 ^a^ | R | 100 | 100 | S | 100 | 58 ^a^ | R | 100 | 100 | S |
|  | Luho | 100 | 60 ^a^ | R | 100 | 100 | S | 100 | 56 ^a^ | R | 100 | 100 | S |
|  | Kessounou | 100 | 71.3 ^a^ | R | 100 | 100 | S | 100 | 25.5 ^a^ | R | 100 | 100 | S |
|  | Savi | 100 | 76 ^a^ | R | 100 | 100 | S | 100 | 62 ^a^ | R | 100 | 100 | S |
| Atlantique-Littoral | Nianouli | 100 | 63 .1 ^a^ | R | 100 | 100 | S | 100 | 39.9 ^a^ | R | 100 | 100 | S |
|  | Sekou | 100 | 84 ^a^ | R | 100 | 100 | S | 100 | 50 ^a^ | R | 100 | 100 | S |
|  | Godomey | 100 | 64 ^a^ | R | 100 | 100 | S | 100 | 38 ^a^ | R | 100 | 100 | S |
|  | Tori | 100 | 75 ^a^ | R | 100 | 100 | S | 100 | 45 ^a^ | R | 100 | 100 | S |
|  | Bohicon | 100 | 80 ^a^ | R | 100 | 100 | S | 100 | 58 ^a^ | R | 100 | 100 | S |
|  | Sehoue | 100 | 72 ^a^ | R | 100 | 100 | S | 100 | 44 ^a^ | R | 100 | 100 | S |
|  | Ze | 100 | 80 ^a^ | R | 100 | 100 | S | 100 | 50 ^a^ | R | 100 | 100 | S |
|  | Suru-Lere | 220 | 75 ^a^ | R | 100 | 100 | S | 300 | 58 ^a^ | R | 100 | 100 | S |
|  | Minontchou | 97 | 64.3 ^a^ | R | 100 | 100 | S | 51 | 56 ^a^ | R | 100 | 100 | S |
|  | Tchankpame | 107 | 60.7 ^a^ | R | 100 | 100 | S | 46 | 25 ^a^ | R | 100 | 100 | S |
|  | Gbedjromede | 112 | 31.3 ^a^ | R | 100 | 100 | S | 25 | 15.2 ^a^ | R | 100 | 100 | S |
|  | Ladji | 90 | 60 ^a^ | R | 100 | 100 | S | 60 | 30 ^a^ | R | 100 | 100 | S |
|  | Fifadji | 128 | 66.4 ^a^ | R | 100 | 100 | S | 59 | 20.3 ^a^ | R | 100 | 100 | S |
|  | Zogbo | 68 | 52.9 ^a^ | R | 100 | 100 | S | 36 | 11.11 ^a^ | R | 100 | 100 | S |
|  | Yenawa | 103 | 71 ^a^ | R | 100 | 100 | S | 40 | 42 ^a^ | R | 100 | 100 | S |
|  | Houeyiho 1 | 100 | 70 ^a^ | R | 100 | 100 | S | 80 | 22 ^a^ | R | 100 | 100 | S |
|  | Houeyiho 2 | 256 | 77 ^a^ | R | 100 | 100 | S | 288 | 40 ^a^ | R | 100 | 100 | S |

(NB. Numbers in the same column with the same superscript do not differ significantly by Fisher’s test (P› 0.05)
