# Supplementary material for: Insecticide resistance status in Anopheles gambiae in southern Benin
Source: Malar J. 2010 Mar 24;9:83. doi: 10.1186/1475-2875-9-83 (PMC2858214; doi:10.1186/1475-2875-9-83)
Supplement: Additional file 2 — Species identification, molecular forms and frequency of the kdr, and Ace.1 alleles and genotypes in Anopheles gambiae s.l [file 1475-2875-9-83-S2.DOC]

**Additional file 2:**

**Species identification, molecular forms and frequency of the *kdr*, and *Ace.1* alleles and genotypes in *Anopheles gambiae s.l.***

|  |  | **Species^a^** | | **Mol. Form** | | ***Kdr mutation*** | | | | ***Ace.1 mutation*** | | | |
| --- | --- | --- | --- | --- | --- | --- | --- | --- | --- | --- | --- | --- | --- |
| **Departments** | **Locality** | **Am** | **Ag** | **M** | **S** | **SS** | **RS** | **RR** | **F(R)** | **SS** | **RS** | **RR** | **F(R)** |
|  | Dogbo | 0 | 30 | 29 | 1 | 0 | 2 | 28 | 0.96** | 29 | 1 | 0 | 0.01 |
|  | Lokossa | 0 | 30 | 27 | 3 | 0 | 3 | 27 | 0.95** | 28 | 1 | 1 | 0.05 |
| Mono-Couffo | Aplahoue | 0 | 35 | 30 | 5 | 0 | 5 | 30 | 0.92** | 35 | 0 | 0 | 0.00 |
|  | Houéyogbé | 0 | 40 | 38 | 2 | 6 | 15 | 19 | 0.67** | 40 | 0 | 0 | 0.00 |
|  | Lanta | 0 | 30 | 25 | 5 | 0 | 3 | 27 | 0.95** | 29 | 1 | 0 | 0.01 |
|  | Klouekanmè | 0 | 35 | 29 | 6 | 4 | 10 | 21 | 0.74** | 30 | 0 | 0 | 0.00 |
|  | Atiémè | 0 | 25 | 22 | 3 | 1 | 7 | 17 | 0.82** | 25 | 0 | 0 | 0.00 |
|  | Toviklin | 0 | 30 | 28 | 2 | 1 | 5 | 24 | 0.97** | 30 | 0 | 0 | 0.00 |
|  | Lalo | 0 | 30 | 29 | 1 | 2 | 8 | 20 | 0.8** | 30 | 0 | 0 | 0.00 |
|  | Possotomè | 0 | 30 | 30 | 0 | 0 | 11 | 19 | 0.81** | 30 | 0 | 0 | 0.00 |
|  | Bopa | 0 | 30 | 27 | 3 | 0 | 9 | 21 | 0.85** | 30 | 0 | 0 | 0.00 |
|  | Comè | 0 | 30 | 27 | 3 | 1 | 11 | 18 | 0.78** | 30 | 0 | 0 | 0.00 |
|  | Pobe | 5 | 25 | 30 | 0 | 6 | 13 | 11 | 0.58** | 30 | 0 | 0 | 0.00 |
|  | Sakete | 0 | 30 | 30 | 0 | 0 | 6 | 24 | 0.90** | 30 | 0 | 0 | 0.00 |
| Ouémé-Plateau | Ifangni | 4 | 36 | 40 | 0 | 38 | 2 | 0 | 0.025* | 40 | 0 | 0 | 0.00 |
|  | Onigbolo | 5 | 35 | 40 | 0 | 35 | 4 | 1 | 0.075** | 40 | 0 | 0 | 0.00 |
|  | Adja-Were | 7 | 23 | 30 | 0 | 1 | 8 | 21 | 0.83** | 30 | 0 | 0 | 0.00 |
|  | Ketou | 0 | 30 | 30 | 0 | 0 | 5 | 25 | 0.91** | 30 | 0 | 0 | 0.00 |
|  | Dangbo | 0 | 30 | 30 | 0 | 0 | 11 | 19 | 0.81** | 30 | 0 | 0 | 0.00 |
|  | Adjohoun | 3 | 27 | 30 | 0 | 4 | 9 | 17 | 0.71** | 30 | 0 | 0 | 0.00 |
|  | Agblangandan | 4 | 26 | 30 | 0 | 29 | 1 | 0 | 0.01* | 40 | 0 | 0 | 0.00 |
|  | Misserete | 0 | 30 | 30 | 0 | 1 | 6 | 23 | 0.86** | 40 | 0 | 0 | 0.00 |
|  | Djeregbe | 4 | 26 | 30 | 0 | 0 | 5 | 25 | 0.91** | 30 | 0 | 0 | 0.00 |
|  | Luho | 7 | 23 | 30 | 0 | 0 | 8 | 22 | 0.86** | 25 | 3 | 2 | 0.11 |
|  | Kessounou | 0 | 30 | 30 | 0 | 0 | 11 | 19 | 0.81** | 30 | 0 | 0 | 0.00 |
|  | Savi | 0 | 30 | 30 | 0 | 6 | 13 | 12 | 0.61* | 30 | 0 | 0 | 0.00 |
|  | Nianouli | 0 | 30 | 30 | 0 | 0 | 10 | 20 | 0.83** | 30 | 0 | 0 | 0.00 |
| Atlantique-littoral | Sekou | 0 | 30 | 30 | 0 | 0 | 4 | 26 | 0.93** | 28 | 2 | 0 | 0.03 |
|  | Godomey | 0 | 30 | 30 | 0 | 0 | 3 | 27 | 0.95** | 29 | 1 | 0 | 0.01 |
|  | Tori | 0 | 30 | 30 | 0 | 1 | 6 | 23 | 0.86** | 40 | 0 | 0 | 0.00 |
|  | Bohicon | 0 | 30 | 28 | 2 | 1 | 9 | 20 | 0.81** | 30 | 0 | 0 | 0.00 |
|  | Sehoue | 0 | 30 | 30 | 0 | 0 | 13 | 17 | 0.78** | 30 | 0 | 0 | 0.00 |
|  | Ze | 0 | 30 | 30 | 0 | 0 | 10 | 20 | 0.83** | 30 | 0 | 0 | 0.00 |
|  | Suru-Lere | 0 | 30 | 30 | 0 | 0 | 5 | 25 | 0.91** | 30 | 0 | 0 | 0.00 |
|  | Minontchou | 0 | 30 | 30 | 0 | 0 | 5 | 25 | 0.91** | 30 | 0 | 0 | 0.00 |
|  | Tchankpame | 0 | 30 | 30 | 0 | 0 | 6 | 24 | 0.90** | 30 | 0 | 0 | 0.00 |
|  | Gbedjromede | 0 | 30 | 30 | 0 | 0 | 10 | 20 | 0.83 | 30 | 0 | 0 | 0.00 |
|  | Ladji | 0 | 47 | 47 | 0 | 1 | 16 | 30 | 0.80 | 47 | 0 | 0 | 0.00 |
|  | Fifadji | 0 | 30 | 27 | 3 | 1 | 11 | 18 | 0.78** | 30 | 0 | 0 | 0.00 |
|  | Zogbo | 0 | 25 | 22 | 3 | 1 | 7 | 17 | 0.82** | 25 | 0 | 0 | 0.00 |
|  | Yenawa | 0 | 30 | 30 | 0 | 0 | 11 | 19 | 0.81** | 30 | 0 | 0 | 0.00 |
|  | Houeyiho 1 | 0 | 45 | 45 | 0 | 1 | 15 | 26 | 0.80 | 42 | 0 | 0 | 0.00 |
|  | Houeyiho 2 | 0 | 30 | 29 | 1 | 2 | 8 | 20 | 0.8** | 30 | 0 | 0 | 0.00 |

^a^ Am, *An. melas*; Ag, *An. gambiae s.s*; ^*^ fully susceptible;  ^**^ fully resistant
